# Arthroscopic Autologous Iliac Crest Bone Grafting for Augmentation of Glenoid Bone Loss Using Suture Anchor Fixation Combined With the Remplissage Procedure

**DOI:** 10.1016/j.eats.2025.103952

**Published:** 2025-11-11

**Authors:** Zhimian Zhang, Xiaobing Xiang, Jianfa Chen, Jie Li, Yuanyuan Wang

**Affiliations:** Department of Sports Medicine, The First Affiliated Hospital of Guangzhou University of Chinese Medicine, Guangzhou, China

## Abstract

For patients experiencing recurrent anterior shoulder dislocation with accompanying glenoid bone loss, glenoid reconstruction via bone grafting is crucial for restoring joint stability. Current fixation methods can be classified into rigid approaches, such as compression screws, and nonrigid alternatives, including suture button plates and suture anchors. While rigid fixation carries risks associated with stress shielding–induced graft resorption and potential screw impingement, nonrigid fixation techniques, such as suture button methods, present their own set of challenges, necessitating specialized instrumentation for precise bone tunnel preparation. This article describes an innovative modification of the Eden-Hybinette technique that employs anchor-based nonrigid fixation. Performed entirely through an intra-articular approach, this method offers dual advantages: it significantly simplifies the surgical procedure while mitigating the complications associated with the retention of permanent metallic hardware in the glenoid.

Glenoid bone loss occurs in 41% of first-time traumatic shoulder instability cases, with the incidence escalating to 86% in instances of recurrent instability.^1^ Isolated soft tissue repair for recurrent anterior dislocation accompanied by concomitant bone loss exhibits notably high failure rates.^2^ Current evidence recommends glenoid bone grafting when bone loss exceeds 25% of the articular surface,^3^ although this threshold is lowered to ≥15% for young athletes engaged in contact sports.^4^ Surgical options include rigid fixation (compression screws) and nonrigid fixation (suture button constructs or anchors), with the former being associated with risks of stress shielding, leading to graft resorption and subsequent osteoarthritis development.^5^ Biomechanical and clinical studies confirm that nonrigid techniques (suture anchors, suture button fixation, and implant-free methods) provide equivalent fixation strength to rigid fixation.^6^ While suture button fixation requires specialized instrumentation for bone tunnel preparation and results in permanent metallic hardware retention, anchor fixation offers technical simplicity, especially when employing bioabsorbable anchors to avoid complications associated with metal implantation. Bone graft sources comprise autografts (iliac crest, distal clavicle, coracoid process, or scapula) and allografts (iliac crest or distal tibia), with allografts having significantly higher resorption rates.^7^

In light of these considerations, we describe a surgical protocol utilizing autologous iliac crest bone graft with suture anchor fixation for glenoid reconstruction in recurrent anterior shoulder dislocation with significant bone loss, showing excellent clinical outcomes.

## Surgical Technique

### General Indications

This technique is recommended for patients exhibiting recurrent anterior shoulder instability with a history of at least 2 dislocations. We consider arthroscopic reconstruction of the glenoid with iliac crest bone graft in cases with glenoid bone loss greater than 20%, in cases with glenoid bone loss between 15% and 20% in younger patients (aged <20 years) involved in competitive or contact sports, and bipolar lesions involving the glenoid and humeral head (Table 1).^8^ Some tips and risks are displayed in Table 1. A summary of the advantages and disadvantages of the procedure is presented in Table 2.^9^

**Table 1:** Indications, Contraindications, Tips, and Risks

**Indications** • Glenoid bone loss >20% • Glenoid bone loss is between 10% and 20% in younger patients (aged <20 years) involved in competitive or contact sports and in bipolar lesions involving the glenoid and humeral head.
**Contraindications** • Patients with multidirectional shoulder instability • Patients with active epilepsy (due to seizure-related reinjury risk)
**Tips** • A 3-dimensional reconstruction should be performed to define the glenoid defect in terms of width and area on the reconstructed en face view of the glenoid. • The unicortical iliac crest graft should be harvested from 3 cm behind the anterior superior iliac spine. The No. 2 Ethibond sutures (Ethicon) passed through holes B and C provide provisional fixation while enabling accurate bone graft alignment during glenoid reconstruction. • The special instruments or a modified 10-mL syringe (with its distal tip removed) should be used to deliver the bone fragment to the desired location.
**Risks** • Avulsion fracture of the spina iliaca • Malpositioning of the graft

**Table 2:** Advantages and Disadvantages

**Advantages of Nonrigid Fixation With Suture Anchors** • Simplified surgical procedure with a gentle learning curve, facilitating technique dissemination • No need for specialized instruments • No bone tunnels required, minimizing scapular bone loss and reducing fracture risk • Nonrigid fixation allows subsequent remodeling of the grafted bone • Avoids hardware complications: no risk of screw breakage, migration, or impingement—common issues with rigid fixation • Dynamic restoration: The 4-point suture configuration (3- and 5-o’clock anchors) adapts to native glenoid curvature during knot tying, preventing overhang. • The all-arthroscopic technique preserves the integrity of the subscapularis tendon, thereby preventing postoperative weakness and loss of external rotation. • Optimal bone block positioning prevents cartilage damage caused by friction and impingement.
**Disadvantages** • Donor site morbidity is possible (iliac crest fracture, nerve injury, gait disturbance, hip pain, sensory disturbance, hematoma/seroma, perforation).^9^ • Suture anchors may lack the initial stability of screws in osteoporotic bone. • It is contraindicated in cases of severe osteoporosis or substantial bone loss. • The all-arthroscopic technique has a steep learning curve.

### Preoperative Planning

**Fig 1:**
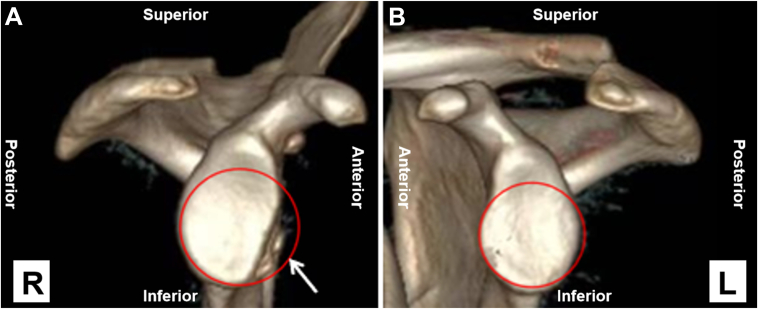
Preoperative 3-dimensional computed tomography scans of the patient's right shoulder, demonstrating the glenoid defect (A), and the normal left shoulder (B). Significant anterior glenoid bone loss is present in the right shoulder (white arrow).

Prior to surgery, routine radiographs and magnetic resonance imaging examinations are conducted. The magnetic resonance imaging examination can exclude patients with humeral avulsion of the glenohumeral ligament. On both sides, computed tomography scans are taken, and 3-dimensional reconstruction is performed to define the glenoid defect in terms of width and area on the reconstructed en face sagittal view of the glenoid (Fig 1). We correctly assess the actual bone defect volume and roughly determine the size of the bone graft according to the length and width of bone loss. The bone graft is generally equal to the amount of bone loss.

### General Preparation

**Fig 2:**
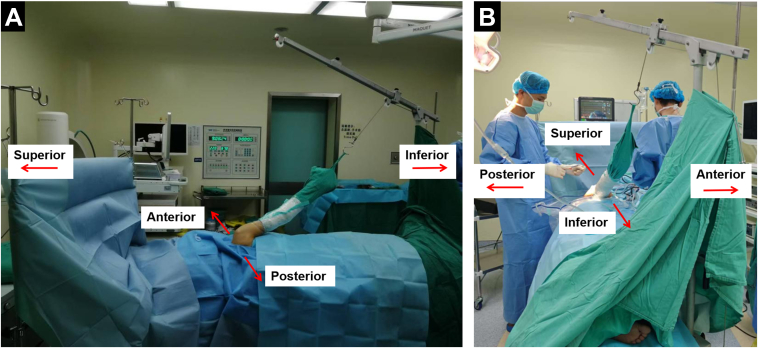
The patient is placed in the lateral decubitus position for a right shoulder arthroscopy, with additional lateral traction. Posterior view (A) and inferior view (B) of the right shoulder.

**Fig 3:**
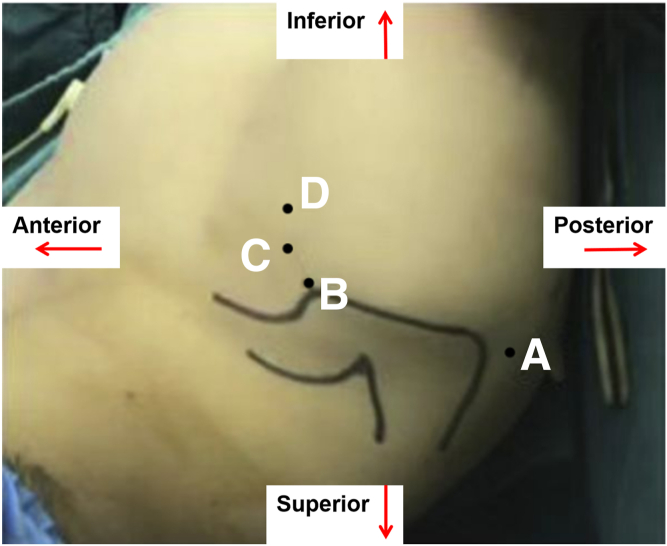
Skin markings for standard arthroscopy portals on a right shoulder: posterior (A), anterosuperior (B), anterior (C), and anteroinferior (D) portals. The image depicts the patient's right shoulder from the superior perspective.

Under an interscalene block and general anesthesia, the patient is placed in the lateral decubitus position with additional lateral traction (Fig 2). Standard shoulder arthroscopy portal sites are marked on the skin (Fig 3), followed by sterile preparation and draping of the operative arm and ipsilateral iliac crest. The arm is secured in a 3-Point Shoulder Distraction System (model AR-1600M; Arthrex) with 45° abduction, 20° anteflexion, and 4 to 5 kg of vertical traction. To ensure adequate blood supply to essential organs, it is advisable to reduce systolic/diastolic blood pressure to around 100/70 mm Hg. Blood pressure should be optimized in hypertensive or elderly patients with comorbidities due to their elevated risk of stroke.

#### Iliac Crest Graft Harvesting and Preparation

**Fig 4:**
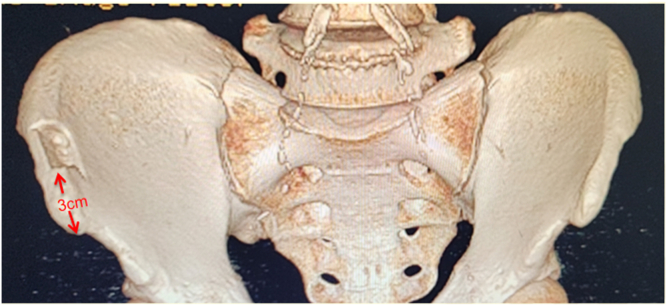
The unicortical iliac crest graft is harvested from the ipsilateral (right) side, starting 3 cm posterior to the anterior superior iliac spine.

The unicortical iliac crest graft is harvested from the ipsilateral side, starting 3 cm posterior to the anterior superior iliac spine (Fig 4).

**Fig 5:**
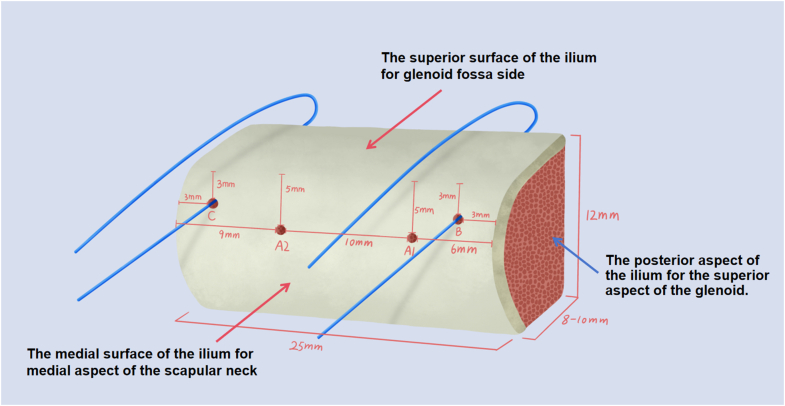
Parameters of the 4 bone tunnels, viewed from the lateral perspective of a right-sided graft. The most commonly used graft is 8-10 mm in width, 25 mm in length, and 12 mm in height, although the size can be customized depending on the case. Four 2 mm diameter holes (A1, A2, B, C) are drilled on the lateral aspect of the bone graft with the following specifications: Holes B and C: 3 mm below the superior surface. Hole B: 3 mm from the posterior border. Hole C: 3 mm from the anterior border. Holes A1 and A2: 6 mm below the superior surface. Hole A1: 6 mm from the posterior border. Hole A2: 9 mm from the anterior border. No. 2 Ethibond sutures are then passed through holes B and C and secured for subsequent use.

The most commonly used graft is 8 to 10 mm in width, 25 mm in length, and 12 mm in height (Fig 5), although the size may be tailored to the specific case. Four 2-mm diameter holes (A1, A2, B, C) are drilled into the lateral aspect of the bone graft according to the following specifications: holes B and C, 3 mm below the glenoid fossa side; hole B, 3 mm from the superior border; hole C, 3 mm from the inferior border; holes A1 and A2, 6 mm below the glenoid fossa side; hole A1, 6 mm from the superior border; and hole A2, 9 mm from the inferior border. No. 2 Ethibond Excel polyester sutures (Ethicon) are then passed through holes B and C and secured for subsequent use.

#### Surgical Portals and Arthroscopic Inspection

**Fig 6:**
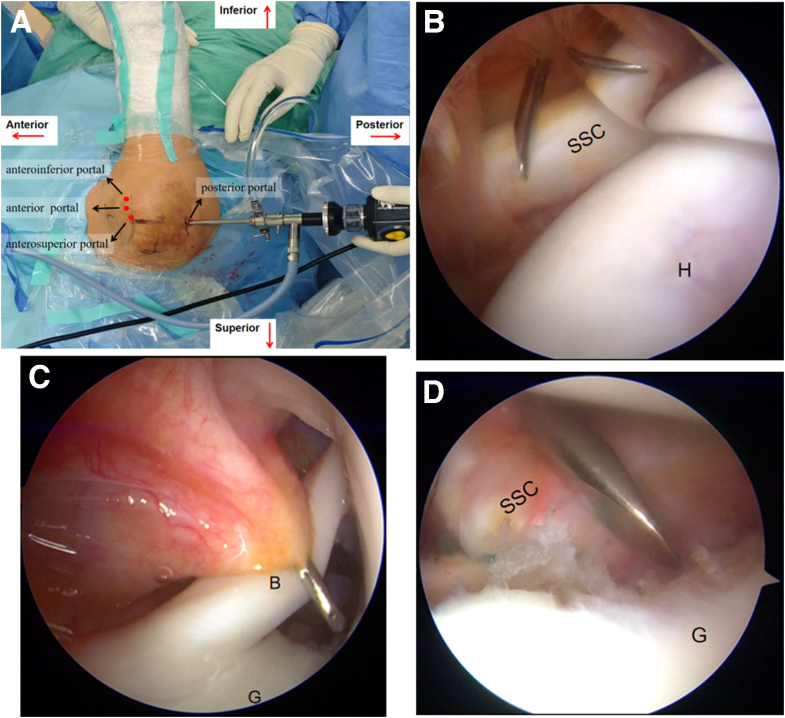
Arthroscopic portal establishment in a right shoulder. (A) The arthroscope is inserted through the posterior portal. (B) Under arthroscopic visualization from the posterior portal, the anterior portal is established in the rotator interval just above the subscapularis tendon. (C) The anterosuperior portal is created posterior to the biceps tendon. (D) The anteroinferior portal (5-o’clock portal) is placed at the superior one-third of the subscapularis tendon.

A standard posterior portal is established for insertion of the 4.0-mm 30° arthroscope (Ref. 72202087; Smith & Nephew). Following this, 1 anterior portal is placed in the rotator interval just above the subscapularis tendon, and the anterosuperior portal is situated behind the biceps tendon. The anteroinferior portal (5-o’clock portal) is placed at the superior one-third of the subscapularis tendon (Fig 6).

The 4.0-mm 30° arthroscope (Smith & Nephew) is introduced through the anterosuperior portal, facilitating further decortication of the anterior glenoid rim and preparation of the glenoid neck with a motorized burr to create a flat, bleeding bony surface.

**Fig 7:**
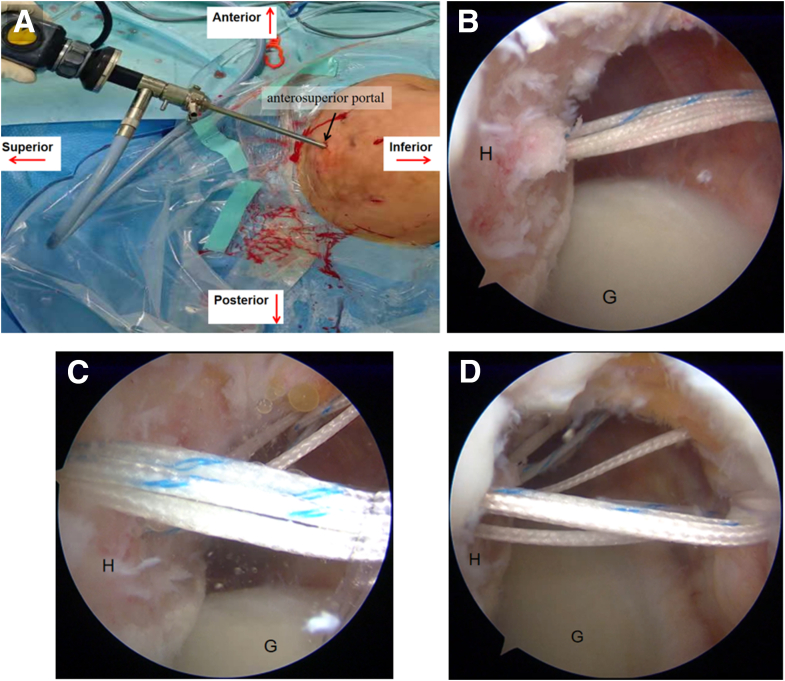
Arthroscopic remplissage technique performed on a right shoulder for off-track bipolar bone loss. (A) Arthroscope positioned through the anterosuperior portal. (B) Placement of inferior anchor at the medial margin of the Hill-Sachs defect. (C) Insertion of the superior anchor 1 to 1.5 cm cephalad to the inferior anchor. (D) Final view showing proper anchor positioning and suture passage through the posterior capsule and infraspinatus tendon. (G, glenoid; H, humerus.)

The arthroscope is then advanced through the anterosuperior portal for diagnostic arthroscopy to evaluate whether the glenoid bone loss constitutes off-track bipolar bone loss according to established criteria.^10^ Upon confirming an off-track lesion, two 4.5-mm double-loaded TWINFIX suture anchors (Smith & Nephew) are inserted at the superior and inferior margins of the central Hill-Sachs defect in the posterosuperior aspect of the humeral head. The sutures from each anchor are passed and left untied for subsequent use (Fig 7). The surgical plan entails securing the infraspinatus tendon into the humeral head defect using a double-pulley suture configuration, with final knot tying to be performed only after completion of the Bankart repair procedure.

**Fig 8:**
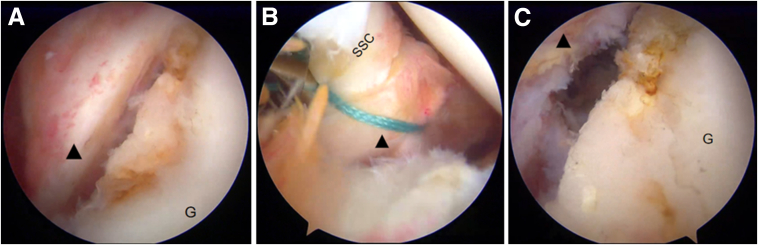
Arthroscopic release of the middle glenohumeral ligament (MGHL) in a right shoulder, under visualization from the anterosuperior portal (as depicted in Figure 7A). (A) The MGHL is routinely released from the anterior glenoid. (B) The MGHL is temporarily suture-tensioned to the superior border of the subscapularis tendon. (C) The anterior capsular space is increased following release. Black triangle, MGHL. (G, glenoid; SSC, subscapularis.)

Following this, the arthroscope is reintroduced through the anterosuperior portal. The middle glenohumeral ligament (MGHL) is routinely released from the anterior glenoid. Once fully released, the MGHL is temporarily suture-tensioned to the superior border of the subscapularis tendon (Fig 8B). This provisional fixation offers 2 advantages: (1) it augments the anterior capsular space, and (2) it mitigates the risk of soft tissue interposition during bone graft placement.

**Fig 9:**
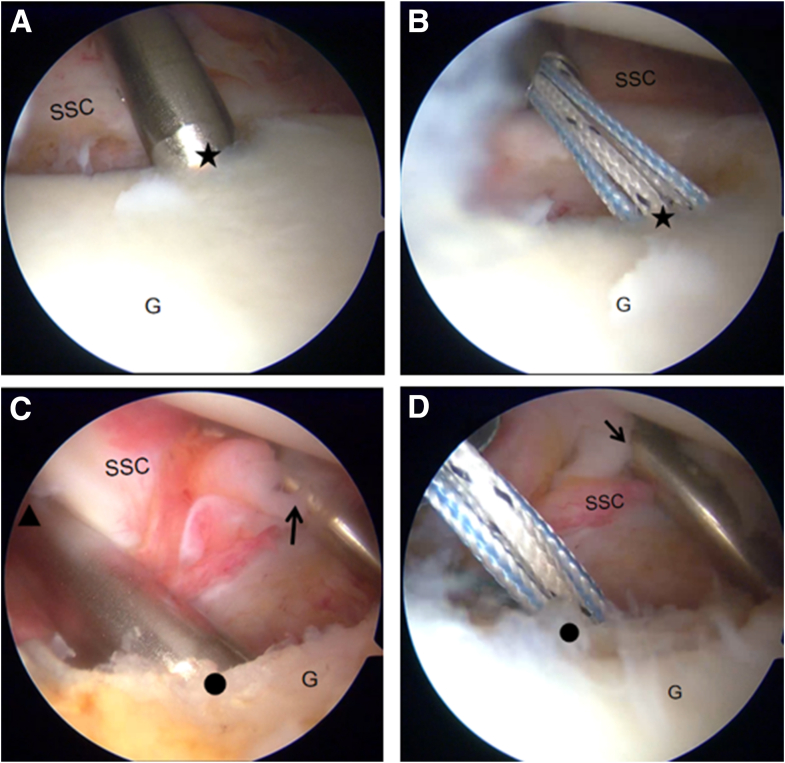
Arthroscopic placement of suture anchors in a right shoulder, under visualization from the posterior portal (as depicted in Figure 6A). (A, B) The 5:00 anchor is positioned at the glenoid rim via the anteroinferior portal. (C, D) The 3:30 anchor is positioned 5 mm below the glenoid surface via the anterior portal. Black arrow, anteroinferior portal; black triangle, anterior portal; black dot, location of the 3:30 anchor; black star, location of the 5:00 anchor. (G, glenoid; SSC, subscapularis.)

A 3.0-mm double-loaded BioComposite SutureTak suture anchor (Arthrex) is first implanted at the 5-o’clock position on the glenoid rim via the anteroinferior portal (Fig 9B). A second 3.0-mm absorbable double-loaded anchor is then placed at the 3:30 position, 5 mm below the glenoid surface, through the anterior portal (Fig 9D). The anchor sutures are retrieved through the posterior portal.

**Fig 10:**
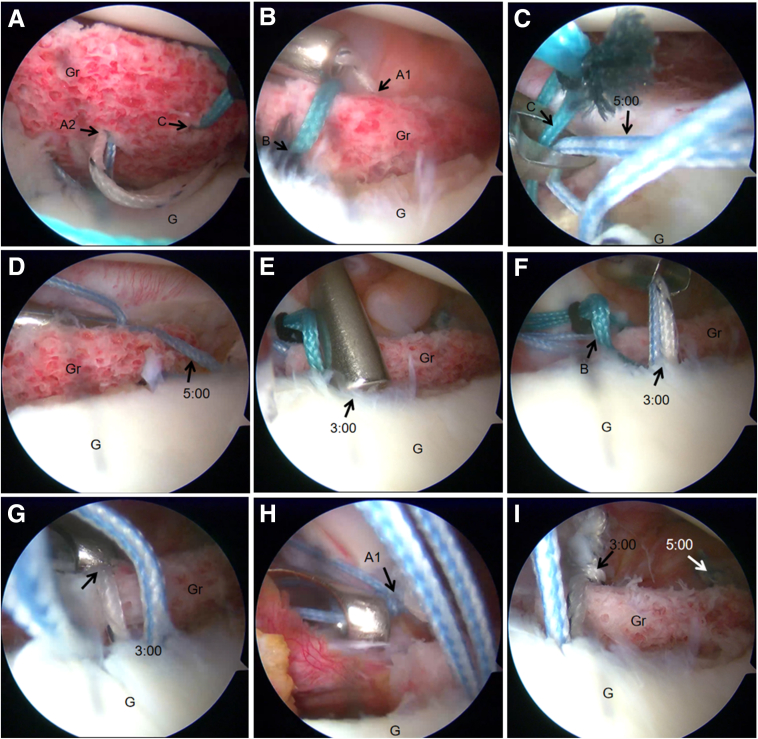
Arthroscopic bone graft fixation in a right shoulder, performed under visualization from the posterior portal (as depicted in Figure 6A). Four suture tails from the 3:30 anchor pass through the A1 hole and A2 hole, respectively. (A) The No. 2 Ethibond suture (Ethicon) is preloaded in hole C. (B) The first fixation is achieved by tying the matched suture pair from the 3:30 anchor, using the A1 hole suture as the post to inferiorly displace the graft during knot tying, providing initial stabilization. (C) One strand from the 5-o’clock anchor is retrieved through the cannula, guiding it through hole C using the preplaced traction suture. (D) The sutures from the 5-o’clock anchor are tied to secure the inferior margin of the bone block. (E) The 3:00 anchor is positioned at the glenoid rim via the anteroinferior porta. (F) One strand from the 3-o’clock anchor is retrieved through the cannula, guiding it through hole B using the preplaced traction suture. (G) The sutures from the 3-o’clock anchor are tied to secure the superior margin of the bone block. (H) The final fixation is completed by tying the remaining suture from the 3:30 anchor while using the A2 hole suture as the post. (I) Postfixation appearance: the B and C holes are securely fixed by sutures from the 3-o’clock and 5-o’clock anchors, respectively. (G, glenoid; Gr, bone graft.)

The anterior portal is extended to approximately 30 mm to accommodate either a specialized bone graft cannula^6^ or a modified 10-mL syringe (with its distal tip removed). Through this cannula, all 4 suture tails from the 3:30 anchor are retrieved, with the differently colored suture pairs separately threaded through the 2 central bone tunnels (A1 and A2) in the iliac bone graft. The prepared bone graft is then carefully delivered through the cannula and positioned anterior to the glenoid neck (Fig 10A). The bone graft is meticulously adjusted to its optimal position with the cancellous bone surface firmly apposed to the anterior glenoid neck. The first fixation is achieved by tying the matched suture pair from the 3:30 anchor, using the A1 hole suture as the post to inferiorly displace the graft during knot tying, providing initial stabilization (Fig 10B).

For inferior border fixation, 1 strand from the 5-o’clock anchor is retrieved through the cannula, guiding it through hole C using the preplaced traction suture, and securely tied (Fig 10D). A subsequent double-loaded anchor is then placed at the 3-o’clock position on the glenoid rim via the anteroinferior portal (Fig 10F). Superior fixation is accomplished by retrieving 1 strand from this 3-o’clock anchor through the cannula, passing it through hole B with the preplaced suture, and tying it securely (Fig 10G).

Final stabilization is achieved by tying the remaining suture pair from the 3:30 anchor that was passed through holes A1 and A2 (Fig 10H), culminating in a 4-point anchor fixation construct that provides multidirectional stability to the bone graft (Fig 10I).

**Fig 11:**
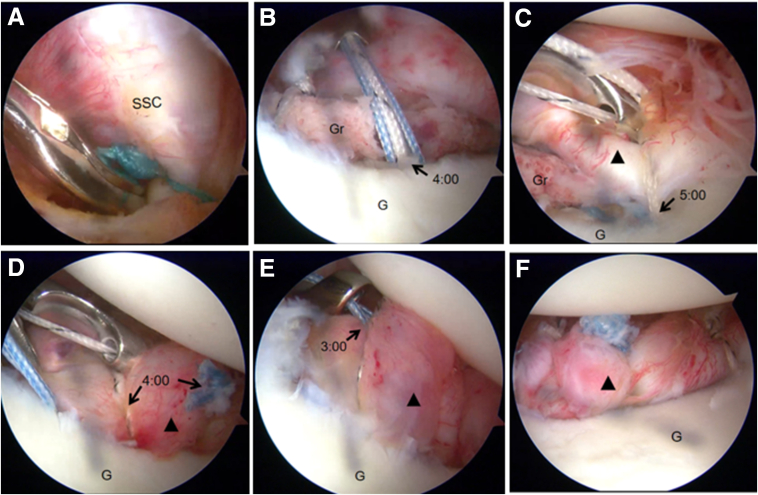
Arthroscopic Bankart repair combined with bone graft in a right shoulder, performed under visualization from the posterior portal (as depicted in Figure 6A). (A) Removal of the provisional sutures securing the subscapularis to the middle glenohumeral ligament (MGHL). (B) The 4-o’clock anchor is positioned at the glenoid rim via the anteroinferior portal. (C) The capsule-labrum structure (black triangle) is draped over the bone graft and attached to the glenoid bone strap using the remaining suture on the 5-o’clock anchor. (D) The 2 sutures of the 4-o’clock anchor are used to repair and secure the MGHL. (E) Repair and secure the MGHL with the remaining suture of the 3:00 anchor. (F) The Bankart repair is completed; the bone graft remains external to the joint, serving as a backup to the repaired capsule-labrum structure. (G, glenoid; Gr, bone graft; SSC, subscapularis.)

The provisional sutures securing the subscapularis to the MGHL are removed (Fig 11A). The last 3.0-mm absorbable double-loaded suture anchor is placed at the 4-o’clock position along the glenoid rim (Fig 11B). The remaining suture groups from both the 3-o’clock and 5-o’clock anchors, along with both suture pairs from the 4-o’clock anchor, are then sequentially passed to meticulously repair the capsulolabral complex. This technique ensures that the bone graft remains external to the joint, thereby providing backup support to the repaired capsule-labrum structure. The entire surgical technique is documented in Video 1, which includes audio narration.

All suture limbs from the 2 posterior anchors are retrieved through the posterior portal. Using the double-pulley technique, the sutures are secured to advance and fixate the infraspinatus tendon into the Hill-Sachs defect, completing the remplissage procedure (Table 3).

**Table 3:** Surgical Technique Pearls and Pitfalls

**Pearls** • Harvesting unicortical iliac bone minimizes the risk of anterior superior iliac spine (ASIS) fracture. • Bone graft harvesting should commence 3 cm posterior to the ASIS to prevent injury to the lateral femoral cutaneous nerve. • The anterior portal is used for anchor insertion 5 mm below the glenoid rim at the 3:30 position, while the anteroinferior portal (5-o’clock portal) is employed for anchor placement along the glenoid rim (3:00, 4:00, and 5:00 positions). This approach optimizes anchor distribution and simplifies suture management. • Prior to bone block insertion, temporary tensioning of the sutures in the subscapularis and middle glenohumeral ligament can expand the anterior glenoid space, facilitating surgical maneuvers and preventing soft tissue interposition during graft placement. • When securing the bone block, using the A1 suture as a post ensures inferior graft positioning. • Positioning holes B and C 3 mm below the anterior graft surface and subsequently tightening their sutures helps restore the normal anteroposterior curvature of the glenoid.
**Pitfalls** • Anchors at the 5-o’clock position on the glenoid must be placed via the anteroinferior portal; using an inappropriate portal may result in anchor perforation or breaching the glenoid bone. • During anchor insertion and suture management, viewing from the posterior portal enhances visualization, helps avoid suture tangling, and facilitates knot tying. • Harvesting an excessively large iliac bone graft should be avoided, as it may lead to challenges in graft insertion, fixation, and potential mismatch with the glenoid contour.

### Rehabilitation

After surgery, the shoulder is immobilized in a neutral external rotation brace for 6 weeks. During this initial period, only scapulothoracic closed-chain exercises are allowed. Full range of motion is encouraged during the second 6-week period, and muscle-strengthening exercises begin from the fourth month.^8^ Patients are cleared to participate in contact activities 6 months after the operation.

## Discussion

The management of recurrent anterior shoulder dislocation, particularly when accompanied by glenoid bone defects, poses significant clinical challenges. Evidence indicates that when glenoid bone loss exceeds 20% of the articular surface, patients undergoing isolated Bankart repair have significantly elevated recurrence rates.^11^ Bone grafting procedures, including the Latarjet technique and its modifications involving coracoid transfer, have become widely adopted clinical solutions. Nevertheless, these surgical interventions carry substantial complication risks, including, but not limited to, nerve injury, hardware failure and migration, graft displacement and resorption, and secondary osteoarthritis.^7^^,^^12^

Free bone grafting techniques confer notable advantages by obviating the neural risks associated with coracoid osteotomy and transfer. When performed arthroscopically, free bone grafting techniques additionally preserve the subscapularis muscle’s integrity, making them an increasingly popular choice for glenoid reconstruction. Commonly used graft options include autologous or allogeneic iliac crest bone, distal clavicle autografts, and distal tibia allografts.^10^ Free bone grafting techniques allow precise graft contouring to match the glenoid defect’s geometry. Autologous iliac crest grafts are particularly preferred, as they eliminate allograft-related risks such as nonunion,^7^ infection transmission, and immune rejection,^13^ establishing them as the most widely utilized graft source.^14^ Despite the description of various graft morphologies (e.g., cylindrical, stepped, J-shaped^15^), comparative studies assessing their clinical outcomes remain lacking.

Irrespective of the grafting technique employed, ensuring secure fixation remains a fundamental prerequisite for successful graft incorporation. While screw fixation (rigid fixation) has been predominantly utilized in previous studies due to its superior mechanical strength, this technique presents considerable technical challenges during arthroscopic procedures.^16^ More importantly, rigid fixation may lead to several detrimental effects: stress shielding–induced excessive graft resorption, hardware impingement, and secondary osteoarthritic changes.^16^^,^^17^ These complications associated with implanted devices represent the most common indications for revision surgery. Consequently, nonrigid fixation methods have emerged as an increasingly investigated alternative in recent research. In addition to the established advantages of arthroscopy, like improved cosmesis, lower infection rates, less scar tissue, expedited discharge, and a quicker rehab process, studies indicate that an all-arthroscopic procedure does not come at the expense of subscapularis function or the structural soundness of its tendon and muscle.^18^

**Fig 12:**
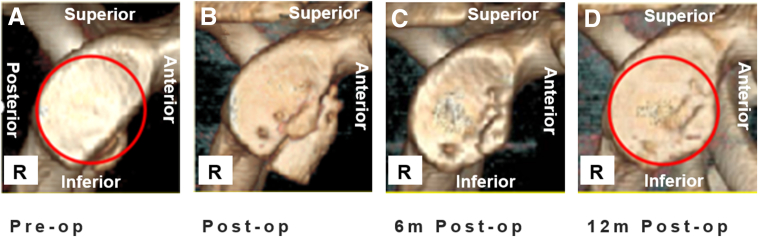
Three-dimensional computed tomography en face views of the right glenoid. (A) Preoperative image. (B) Postoperative image. (C) Image at 6 months, showing complete bone healing and partial remodeling. (D) Image at 12 months, showing complete bone healing and remodeling.

We present an innovative surgical technique for anterior shoulder instability with glenoid bone loss, utilizing an autologous iliac crest graft and a nonrigid fixation system using suture anchors. The procedure involves creating 4 precisely positioned bone tunnels in the graft for multipoint fixation, thereby eliminating the need for specialized glenoid tunnel instrumentation while maintaining fixation reliability and simplifying surgical steps. Compared to the technique by Zhao et al.,^6^ this 4-point fixation construct shows superior biomechanical stability: the suture systems from the 3- and 5-o’clock anchors facilitate dynamic restoration of the native glenoid curvature during knot tying, effectively preventing graft protrusion beyond the articular surface and consequently reducing osteoarthritis risk.^19^ Postoperative evaluations confirm that the nonrigid fixation allows for stress-mediated remodeling of the graft, ultimately achieving near-anatomic restoration of the pear-shaped glenoid morphology (Fig 12). Combined with repair of the glenohumeral ligament complex (Bankart lesion), the compressive forces exerted by the subscapularis, glenoid, and joint capsule synergistically enhance fixation stability and promote graft incorporation.^6^

The optimal graft position requires the majority of the graft to be positioned below the glenoid equator in the coronal plane and 2 to 5 mm medial to the articular surface in the axial plane. Graft malposition is characterized by the majority of the graft being either above the glenoid equator or protruding beyond the articular surface.^20^ During graft preparation, the A1 hole is placed 6 mm from the superior border and the A2 hole 9 mm from the inferior border. The anchor for A1 and A2 sutures is positioned at the 3:30 position on the glenoid, 5 mm medial to the articular surface. Using the A1 suture as a post during knot tying guarantees that the graft is placed inferiorly below the equator. With both A1 and A2 holes positioned 6 mm medial to the graft’s glenoid fossa side, the graft remains parallel to the articular surface when sutures are tightened. Simultaneously, the B and C holes, positioned 3 mm from the anterior surface, facilitate the restoration of the glenoid’s anteroposterior curvature upon tying their corresponding sutures.

Complications arising from autogenous iliac bone graft harvesting include iliac crest fracture, nerve injury, gait disturbance, hip pain, sensory disturbance, hematoma/seroma, and perforation.^9^ Iliac crest bone graft harvesting is associated with a notable range of complications, with reported incidence rates varying from 2% to 49%.^21^ These include minor to severe issues such as persistent donor site pain, cosmetic deformities from contour depression, and scarring. More serious risks encompass gait disturbance, hip pain, sensory deficits, hematoma, seroma, peritoneal perforation, and even “landslide” hernia, particularly after full-thickness graft retrieval.^9^ Pain is frequently severe enough to disrupt sleep within the first month, and 13% to 20% of patients develop chronic pain.^22^ To mitigate these risks, our technique utilizes a monocortical harvesting approach to extract bone from the medial cortex while preserving the integrity of the pelvic ring, thereby averting secondary fractures. The harvest site is positioned 3 cm posterior to the anterior superior iliac spine, with careful subperiosteal dissection along the medial aspect of the ilium to avoid injury to the lateral femoral cutaneous nerve. The fixation technique proposed in this study has significant advantages over suture button fixation: operationally, it eliminates the need for specialized bone tunnel drilling instruments, thus simplifying surgical procedures. In terms of safety, it mitigates the risk of bone loss and fractures associated with tunnel drilling; biomechanically, by eliminating posterior knot tying with suture button fixation, it avoids soft tissue entrapment and improves joint mobility.

The arthroscopic nonrigid anchor fixation technique enables minimally invasive reconstruction of anterior glenoid defects. For cases with greater than 20% glenoid bone loss, we preferentially employ this arthroscopic approach. However, there are limitations to the technique: donor site complications may occur, including iliac crest avulsion fractures and skin numbness at the harvest site.

## Disclosures

All authors (Z.Z., X.X., J.C., J.L., Y.W.) declare that they have no known competing financial interests or personal relationships that could have appeared to influence the work reported in this paper.

## Supplementary Data

Video 1 Arthroscopic autologous iliac crest bone grafting for augmentation of glenoid bone loss using suture anchor fixation combined with the remplissage procedure.
